# Analysis of taste and non-volatile compound differences in jujube pigments from different sources using multiple techniques

**DOI:** 10.1016/j.fochx.2026.104378

**Published:** 2026-08-28

**Authors:** Junlan Li, Shunmeng Chen, Yuchun Zhou

**Affiliations:** a Institute of Rural Revitalization, Hexi University, Zhangye, Gansu 734000, PR China; b College of Agriculture and Ecological Engineering, Hexi University, Zhangye, Gansu 734000, China; c Jiangsu Food & Pharmaceutical Science College, Huaian, Jiangsu 223300, China

**Keywords:** Jujube pigment, Scanning electron microscopy, Electronic tongue, Taste evaluation, UPLC-Q-TOF-MS/MS, Taste-active compounds

## Abstract

While jujube pigment color properties are well documented, its full flavor profile and specific non-volatile constituents remain understudied. Jujube pigments from three sources—jujube production waste residue (BJP), fresh jujubes (FJP), and dried jujubes (DJP)—were compared for non-volatile compounds and flavor profiles. Using SEM, electronic tongue, and UPLC-Q-TOF-MS/MS. SEM showed BJP thin – flaky, FJP/DJP blocky (FJP dispersed, DJP compact); BJP better water - soluble. All three share five core tastes—umami, saltiness, bitterness, sweetness, and aftertaste-B—with FJP strongest in umami, saltiness, and bitterness, and BJP highest in sweetness. UPLC-Q-TOF-MS/MS tentatively identified 25 compounds, including flavonoids, phenolic acids, phenols, and anthraquinones; PLS-DA highlighted 13 key differential compounds. Bitterness, aftertaste-B, umami, richness, and saltiness correlated significantly with caffeic acid, 5-hydroxy-3′,4′,7-trimethyloxyethanol, syringic acid, and quercetin; sweetness correlated with trans-4-coumaric acid and quercetin-3-O-neohesperidin—these six compounds are candidate biomarkers. Findings clarify composition/sensory differences, pinpoint key components, support future research.

## Highlights

• Jujube pigment taste profile: umami, bitterness, bitter aftertaste.
• Jujube pigment contains flavonoids, phenolic acids, anthraquinones, phenols.
• Jujube pigment taste profile correlates significantly with non-volatile substances.
• Jujube peel, a by-product, has high pigment recovery and utilization value.

## 1 Introduction

Color serves as a crucial indicator for assessing the sensory quality of food. It has the potential to stimulate the secretion of digestive juices, enhance appetite, and attract consumers (Manzoor et al., 2021). To enhance the color of products and improve sensory quality, food coloring is often added during the food - processing stage to make up for the color deficiency. Food colorings can be divided into two main categories: synthetic colorings and natural colorings. Synthetic pigments are primarily formed from chemical products, such as benzene, toluene, and naphthalene, through a series of organic reactions, including sulfonation, nitrification, and halogenation. They can be classified into two types of chemical structures: azo and non - azo. (Feng, Cerniglia and Chen, 2012). Prolonged, low - dose intake of synthetic food colorings as food additives may induce gene mutations and cancer (Sharma et al., 2021; Iheanyichukwu, Uwaezuoke and Amanda, 2019; Fried et al., 2022). Nevertheless, synthetic colorings possess high stability, strong coloring capacity, vivid colors, low cost, resistance to acids and alkalis, and ease of color matching, and are extensively utilized in the food industry (Affat, 2021; Prabowo et al., 2022). Consequently, their dosages and application scopes are clearly and systematically regulated by the Codex Alimentarius Commission (CAC). In comparison with synthetic pigments, natural food pigments are characterized by a wide variety of types, abundant sources, low toxicity, high safety levels, and soft colors. Natural pigments obtained through the extraction and purification of animal and plant tissues are categorized as Class A additives by the CAC and are exempt from certification as food pigments. There are no restrictions regarding their usage scope or dosage. Their most prominent advantage lies in the fact that they can be safely degraded (Fried et al., 2022), exhibit a harmonious and natural color, and also contain anti - cancer, antioxidant, and antibacterial bioactive components (Lekshmi et al., 2023; Wang, 2024). They will be an indispensable additive for products in the fields of food, medicine, cosmetics, etc.

The Jujube tree *(Zizyphus jujuba* Mill.), a perennial plant of the Rhamnaceae family, is abundant in nutrients and mineral elements and is a kind of fruit that can serve both as medicine and food. China is the world's leading producer of jujubes, and 90% of these jujubes are preserved or consumed in a dried form (Suzie et al., 2014). The processed products of dry jujube encompass candied dates, jujube paste, red date cakes, beverages, red date wine, red date vinegar, etc. During the processing of jujube products, a large amount of waste residue is produced, which contains certain quantities of jujube pulp, jujube peel, and jujube pit. These waste residues are usually utilized as feed or landfilled as waste. This practice not only imposes a burden on enterprises but also results in resource wastage and environmental pollution. Red jujube peels are rich in cellulose, which cannot be digested by the human body. Nevertheless, the red pigments on their surface have a pleasant aroma and an appealing color (Shen et al., 2021). Prior research has indicated that the content of secondary metabolites in jujube peels was notably higher than that in the flesh and seeds. Studies on the extraction and composition of peel pigments have mainly focused on pigment alterations during the fruit's coloring phase, showing that the principal components of fresh jujube peel pigments were phenolic compounds and their derivatives (Nascimento, et al., 2020; Kandemir et al., 2022; Kou et al., 2019). It is widely recognized that phenolic compounds serve as the primary source of antioxidants and possess anti - inflammatory and anti - cancer properties. These properties can contribute to the reduction of health risks, including hypertension, obesity, cardiovascular diseases, diabetes, and various types of cancer.

To date, studies have reported the aroma compounds in mature fresh jujubes of different varieties (Chen et al., 2018; Galindo et al., 2015; Hernandez et al., 2016) and in dried jujubes processed by various drying methods (Song et al., 2020; Zhu and Xiao, 2018; Wang et al., 2018; Qiao et al., 2023; Song et al., 2022). All these studies used gas chromatography - mass spectrometry (GC - MS) to identify the volatile aromatic components in red jujubes. However, there has been no systematic report on the color and flavor characteristics of pure jujube peel extract. Only a few studies (e.g., Shen et al., 2023; Dou et al., 2023) have used ultra - performance liquid chromatography - tandem mass spectrometry (UPLC - MS/MS) to identify that jujube pigments mainly consist of various phenolic compounds. Nevertheless, these studies have not explored the fundamental active components of jujube pigments in sufficient depth. This significantly hinders the accumulation of scientific data required for the high - value utilization of jujube processing by - products and restricts the practical application and industrial promotion of this pigment. This study employed ultra - performance liquid chromatography - quadrupole time - of - flight mass spectrometry (UPLC - Q - TOF - MS) in combination with scanning electron microscopy (SEM) and electronic tongue - based intelligent sensory evaluation methods to systematically analyze the surface morphology, taste, and non - volatile components of jujube pigments from different sources. The flavors are characterized to elucidate the differences in non - volatile effective components of jujube pigments from diverse sources. This, in turn, offers a theoretical basis for explaining the unique flavor of jujube pigment and its application as a natural edible pigment with both food and medicinal properties.

## 2 Materials and methods

### 2.1 Reagents and equipment

Mass spectrometry-grade methanol, formic acid, and acetonitrile were obtained from CNW Technologies (Germany); Chromatographic grade methanol and acetonitrile were obtained from Sigma-Aldrich (Germany). All the other reagents were of domestic AR grade. Laboratory water was purified using a Milli-Q ultrapure water system. D155 microporous weakly acidic cation exchange resin was purchased from Hecheng New Materials Technology Co., LTD.

SA402B electronic tongue (INSET Corporation, Japan). The InfinityII1290 UPLC was combined with the 6546 A Q – TOF - MS/MS system, which comprised an electrospray ionization (ESI) source, a diode array detector (DAD), an automatic, a column oven, and Mass Hunter software. This system was manufactured by Agilent Technologies, Inc. (USA). The ML204T/02 analytical balance (readability ±0.0001 g) was purchased from Mettler-Toledo (Switzerland). The KQ-500DE ultrasonic cleaner (not CNC-type) was produced by Kunshan Ultrasonic Instrument Co., Ltd.

### 2.2 Experimental material

The experimental materials consisted of peels from locally cultivated grown red jujubes of the same cultivar (*Ziziphus jujuba* Mill. cv. ‘Linzexiaozao’), which were sourced from three different origins. Source one was the jujube waste residue generated during the production and pressing processes of Gansu Xiyu Food Co., Ltd. The waste residue should be repeatedly washed and rubbed with tap water to thoroughly separate the jujube flesh, pits, and peel. Initially, it is filtered through a coarse sieve to remove the jujube flesh. After the remaining jujube pits and peels are naturally air-dried, the pits are removed by sieving. The resulting peel is then ground and crushed for subsequent use. Source two: Procure ripe, fresh jujubes from the market. Clean off surface dust, pare away 2–3 mm of the outer layer, and collect the jujube skins. Then dry and crush them, and store for subsequent use. Source three: For dried jujubes procured from the market, wash and soak them for approximately one hour. Then place them in a steamer filled with room - temperature water and steam for 30 to 40 min. Once removed from the steamer, promptly peel the skins off manually and collect the dried, crushed jujube peel for future use.

### 2.3 Sample preparation

The preparation method of jujube peel pigment extract was modified based on the method described by Wang et al. (2019). A 0.2 mol/L KOH solution was used as the extraction solvent. The extraction temperature was set at 60 °C, the extraction time was 10 h, and the solid - to - liquid ratio was 1:20 (*w*/*v*). After extraction, the residue was separated from the extract by filtration and then the filtrate was passed through a 300 - mesh 304 stainless steel screens. The resulting filtrate was designated as the jujube pigment extract, with a pH of approximately 13.80 and an electrical conductivity of 7884 μs/cm. The pigment solution extracted from the waste residue of jujube beverage production is designated as BJP. The pigment solution extracted from fresh jujube peels is designated as FJP. The pigment solution extracted from the dried peels is designated as DJP.

In the jujube pigment extract solutions of BJP, FJP, and DJP, the microporous weakly acidic cation-exchange resin D155 was added separately at a resin-to-extract volume ratio of 1:30. The sample solutions underwent multiple static desalination treatments until the pH of the jujube pigment extract reached 5.50 and the electrical conductivity approximately 1413 μs/cm; meanwhile the absorbance decreased from 74.9 ± 12.3 to 67.8 ± 9.36 IU/g, correspondting to a pigment loss rate of approximately 9.47%. Subsequently, the jujube pigment solutions of jujube obtained from three distinct sources were poured into a flat-bottomed culture dish with a solution layer thickness of approximately 3 to 5 cm. The dish was then placed in a drying oven maintained at 25 °C until complete drying. Finally, the dried pigment powder was collected as experimental material for subsequent use.

### 2.4 Microscopic structure observation of SEM

Take a small amount of jujube pigment powder from different source, evenly disperse it on the sample stage, and secure it with conductive tape. Remove excess particles using a dust blower. Sputter-coat the sample with gold twice at 5 mA for 30 s each. Then image the samples in an SEM at 15 kV and 5000× to observe the surface microstructure.

### 2.5 Electronic tongue detection

Take 120 mL of jujube pigment solution with a desalinated pH ranging from 4.5 to 5.5, a conductivity of approximately 1413 μS/cm, and an absorbance value of approximately 67.8 ± 9.36 IU/g. Then, directly analyze the flavor profiles of jujube pigments from different sources using the SA402B electronic tongue system. The sensor array consists of six sensors (AAE, CT0, CA0, C00, AE1, GL1, BT0), and the corresponding sensitive tastes are umami, saltiness, sourness, bitterness, astringency, sweetness, and after - bitterness respectively. The ambient temperature for detection was room temperature. Prior to data collection, sensor activation and calibration were performed on the electronic tongue to ensure the reliability and stability of the detection data. A reference solution was prepared by mixing a 30 mM KCl solution with a 0.3 mM tartaric acid solution. The cleaning solution for the counter electrode consists of water, ethanol, and HCl, while that for the working electrode consisted of KCl, water, ethanol, and KOH. The sensor was immersed in the reference solution for zero-setting for 30 s, after which measurement of taste indicators was initiated. The measurement duration was 30 s. After completion of the test, the sensor was rinsed with the reference solution for 3 s and then re-measured. The measurement duration remained 30 s. Each sample was biologically replicated 4–5 times. For analysis, the average of the last three stable response values from the electronic tongue was used.

### 2.6 UPLC-Q-TOF-MS /MS analysis

#### 2.6.1 Preparation of the test solution

Accurately weigh 0.01 g of jujube pigment powder and transfer it into a 50 mL conical flask fitted with a stopper. Precisely add 10 mL of methanol, shake the flask thoroughly, weigh the flask and its contents, subject it to ultrasonic treatment (500 W, 40 kHz) for 30 min. Then it from the ultrasonic bath and allow it cool to room temperature. Adjust the volume with methanol to compensate for solvent loss during sonication, and filter the solution through a 0.22 μm microporous membrane to obtain the test solution.

#### 2.6.2 Chromatographic condition

chromatographic column, ACQUITY UPLC BEH C_18_ (100 mm × 2.1 mm,1.8 μm). mobile phase A: 0.1% formic acid aqueous solution, mobile phase B: acetonitrile, column temperature 35 °C,flow rate 0.25 mL/min, measure wavelength 360 nm, sample size 2.0 μL, gradient elution: 0–15 min, 70% A, 30% B; 15–30 min, 70% ∼ 5% A；30% ∼ 95% B；30–32 min, 5% A, 95% B.

2.6.3 MS condition: A dual-spray atmospheric pressure electrospray ion source was employed (Dual AJS ESI), positive and negative ion mode scanning. Nebulizer: 35Psig; sheath gas temperature: 350 °C; sheath gas flow: 11 L/min; nozzle voltage: 1000 V; Fragmentor: 120 V; use fixed collision energies:35 V; The first – level mass spectrometry data acquisition was performed in the full - scan mode of unipolar mass spectrometry, with a scanning range of *m*/*z* from 100 to 1500 and an acquisition rate of 1 spectrum per second, The secondary mass spectrometry data acquisition operates in an automatic acquisition mode, featuring a scanning range of *m*/*z* from 50 to 1500 and an acquisition rate of 1 spectrum per second.

#### 2.6.3 Precision test

The pigment test solution was injected six consecutive times under the chromatographic conditions described in Section 2.6.2. Using the caffeic acid peak in Sample No. 0 as the reference peak, the relative retention times and relative peak areas of each common peak were calculated. The RSD values were 0.03%–1.23% and 0.11%–2.47%, respectively, indicating good instrument precision.

2.6.5 Stability test: The pigment test solution was sampled and analyzed by HPLC using the chromatographic conditions described in Section 2.6.2 at 0, 4, 8, 12, 18, and 24 h after storage at room temperature. Using the caffeic acid peak of the sample labeled “No. 0” as the reference peak, the relative retention times and relative peak areas of the common peaks were calculated. The RSD values were 0.02%–0.81% and 0.03%–2.96%, respectively, indicating that the test solution remains stable for up to 24 h.

#### 2.6.4 Repeatability test

Six aliquots of the same batch of pigment sample were prepared, and test solutions were prepared according to the procedure described in Section 2.6.1. The samples were injected for chromatographic analysis. Using the chromatographic peak of caffeic acid in Sample No. 0 as the reference peak, the relative retention times and relative peak areas of each common peak were calculated. The RSD values were 0.08%–0.19% and 0.77%–2.76%, respectively, indicating that the method exhibits good repeatability.

### 2.7 UPLC-Q-TOF-MS/MS data processing

Using the Mass Hunter 10.0 data – processing software and relying on the precise relative molecular mass, isotope abundance ratio, and secondary mass spectrometry information provided by high - resolution mass spectrometry, in conjunction with databases such as METLIN, HMDB, MassBank, PubChem, and the component database of Agilent Chinese medicine reference substances, the quasi - molecular ion peaks and fragmentation patterns of compounds in the database were compared and analyzed. The analysis takes into account a mass deviation of ≤3–5 ppm, a database match score of ≥85, and an isotope abundance ratio deviation of fragment base peaks of less than 20%. Subsequently, the chemical components in the sample were tentatively identified, including compound name, molecular formula, exact molecular mass, primary and secondary characteristic fragment ions, and chemical class.

## 3 Results and discussion

### 3.1 The microstructure of jujube pigment powder with different sources

**Fig. 1:**
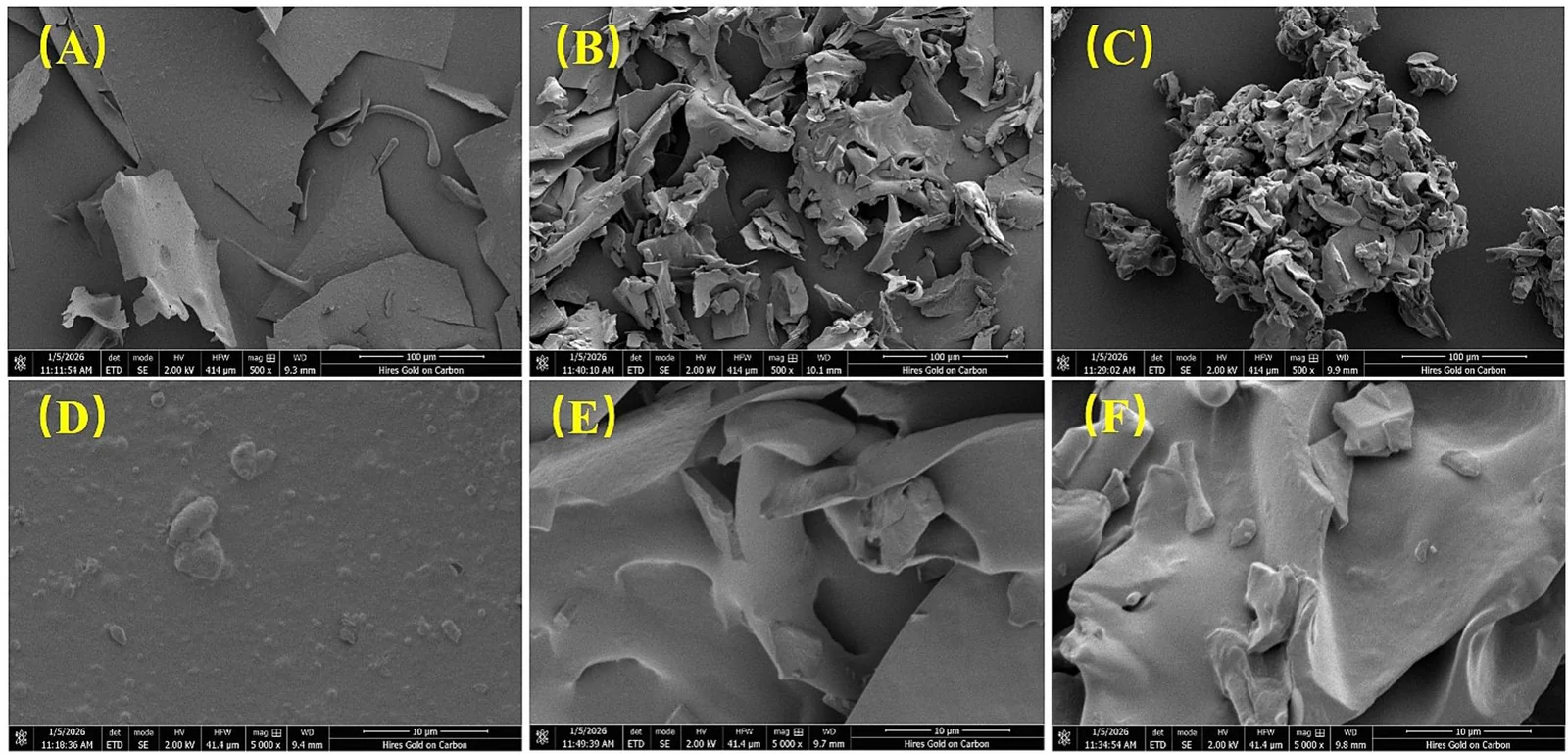
Scanning electron microscopic observation of the surface microstructure of jujube pigment from different sources. (A) and (D) BJP; (B) and (E) FJP; (C) and (F) DJP.

The morphological characteristics of jujube pigment powder from different sources are presented in Fig. 1. At a resolution of 100 μm and a magnification of 500×, BJP(A) exhibits a thin, sheet - like structure. When the magnification was further increased to 5000× [as depicted in Fig.(D)], small plate - like particles were observed attached to the thin, sheet - like matrix. Panel (B) and (C) illustrate the morphologies of FJP and DJP, respectively, both displaying a blocky structure, however, FJP was more dispersed, whereas DJP exhibits a compact structure. Panels (E) and (F) show the morphologies of FJP and DJP, respectively, at 5000× magnification, revealing thick and irregular, sheet - like tissues. It was therefore inferred that BJP exhibits higher water solubility.

### 3.2 Analysis of the taste flavor of jujube pigments using an electronic tongue

**Fig. 2:**
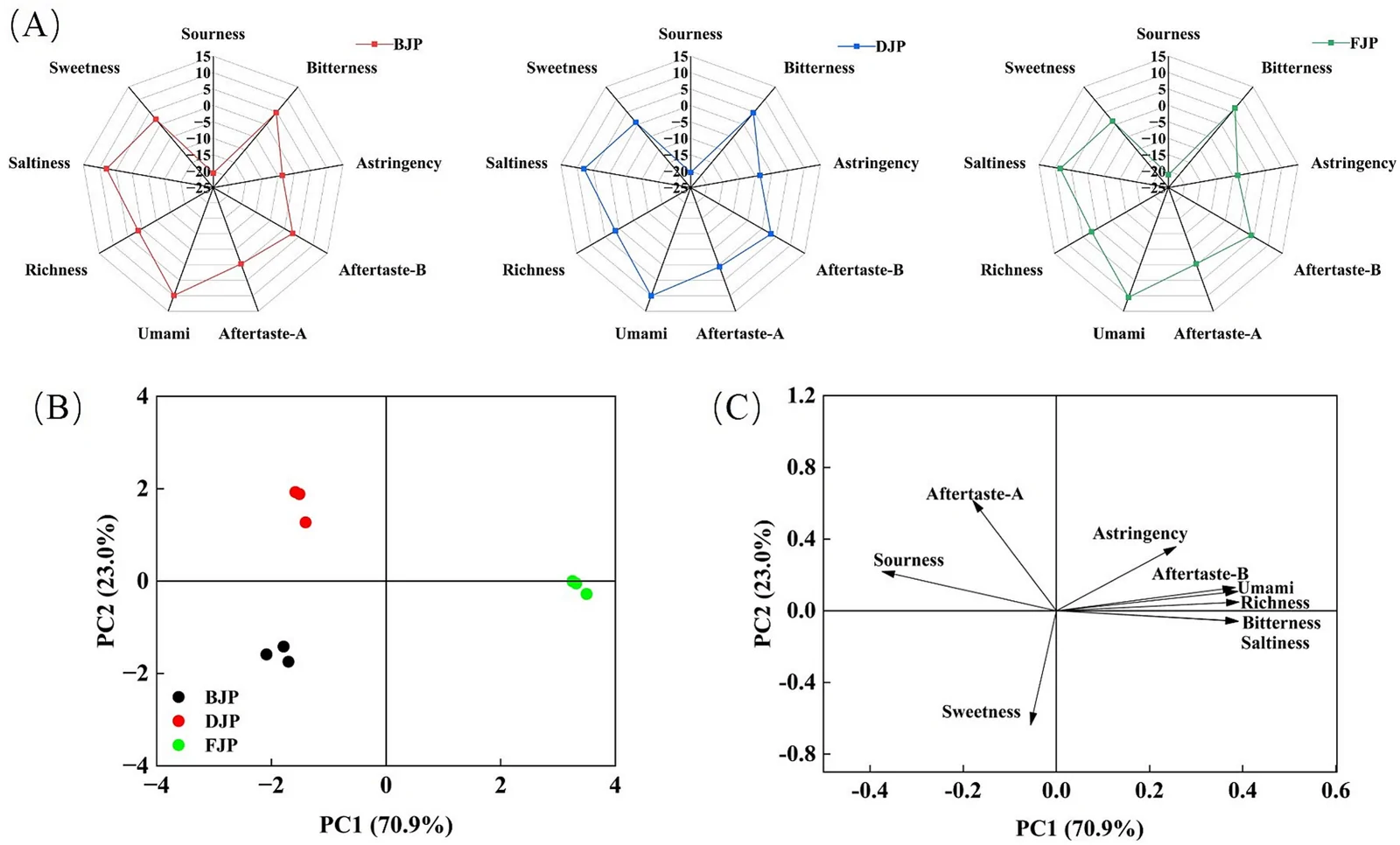
Electronic tongue analysis of jujube pigment taste from different sources. (A)Radar，(B)PCA，(C)Loadings.

The electronic tongue is an intelligent sensory detection technology that mimics human taste perception and can detect the taste characteristics of samples objectively, rapidly, and in a batch - wise manner (Chen et al., 2023; Wesoły et al., 2023; Ghasemi-Varnamkhasti et al., 2018). In this study, by identifying the response intensity of samples using different sensors, seven flavor attributes of jujube pigment, namely sweetness, sourness, umami, astringency (with an astringent aftertaste-A), bitterness (with a bitter aftertaste-B), richness (with a umami aftertaste), and saltiness, were detected. The taste radar charts of jujube pigment samples from different sources are established, as shown in Fig. 2(A), and the taste intensity response values are presented in Table 1. The radar diagram of the electronic tongue indicates that the taste profiles of the three types of jujube pigments, namely BJP, FJP, and DJP, were fundamentally similar. The intensity ranges of umami, saltiness, bitterness, and sweetness were 9.93–10.51, 7.87–8.42, 4.67–6.53, and 0.90–2.11, respectively. Among these tastes, umami, saltiness, and bitterness were the most prominent in FJP. In contrast, BJP was characterized by a more prominent sweetness and exhibited the lowest intensity in umami and saltiness. Aftertaste, which refers to the persistence and richness of the taste, was closely associated with the content of taste substances and the different sources of jujube pigments. The intensity ranges of aftertaste-B and richness aftertaste were 2.88–4.08 and 1.26–1.92, respectively. Among these samples, the FJP exhibited the highest intensity, whereas the BJP had the lowest intensity. The sourness, astringency, and aftertaste-A of BJP and FJP all showed negative values. This phenomenon occurs because the negative response is attributed to the differential measurement mechanism of the electronic tongue. When the concentration of the taste - related substance in the sample is lower than that in the reference solution, the potential difference is registered as a negative value, this region corresponds to the taste intensities undetectable by humans (Tahara and Toko, 2013), indicating insufficient organic acid content and that astringent substances have not yet reached the sensory threshold. Umami, saltiness, aftertaste, aftertaste - B, and sweetness were the important taste characteristics of jujube pigments. According to the reports of Dou et al., 2023 and Pu et al., 2018, jujubes contain polyphenols. Based on this finding, it can be inferred that the saltiness, aftertaste, and aftertaste - B in the taste of jujube pigments may be associated with the types and contents of polyphenols.

**Table 1:** Response values of the electronic tongue to jujube pigments from different sources.

Nomber	Reference	Taste type	Sensor type	Response value		
				BJP	DJP	FJP
1	−13	Sourness	CA0	−20.60 ± 0.02^b^	−20.41 ± 0.02^a^	−21.11 ± 0.03^c^
2	0	Bitterness	CO0	4.75 ± 0.05^b^	4.67 ± 0.09^b^	6.53 ± 0.17^a^
3	0	Astringency	AE1	−3.68 ± 0.05^b^	−3.58 ± 0.04^a^	−3.54 ± 0.06^a^
4	0	Aftertaste-B	BT0(Bitter aftertaste)	2.88 ± 0.12^c^	3.19 ± 0.06^b^	4.08 ± 0.11^a^
5	0	Aftertaste-A	AE1(Astringent aftertaste)	−0.30 ± 0.01^b^	0.64 ± 0.07^a^	−0.31 ± 0.05^b^
6	0	Umami	AAE	9.93 ± 0.01^c^	10.06 ± 0.02^b^	10.51 ± 0.03^a^
7	0	Richness	AAE (Umami aftertaste)	1.26 ± 0.06^b^	1.36 ± 0.07^b^	1.92 ± 0.03^a^
8	−6	Saltiness	CT0	7.89 ± 0.04^b^	7.87 ± 0.03^b^	8.42 ± 0.02^a^
9	0	Sweetness	GL1	2.11 ± 0.33^a^	0.90 ± 0.33^b^	1.38 ± 0.29^b^

Note: Distinct letters signify statistically significant disparities among the samples. (*p*<0.05).

To further clarify the similarities and differences among pigments derived from different sources, principal component analysis (PCA) was performed based on the response values of various sensors of the electronic tongue. As shown in Fig. 2(B), the response values of the tastes of the three jujube pigments, namely BJP, DJP, and FJP, on different sensors indicate that the contribution rates of the first principal component (PC1) and the second principal component (PC2) were 70.9% and 23.0% respectively, with a combined contribution rate of 93.9%. This result effectively captures most of the taste - related information characteristics of the samples in each group. The distance between sample patterns represents the magnitude of taste disparities among samples. In the principal component analysis (PCA) spatial distribution, the three samples, namely BJP, DJP, and FJP, do not overlap and are distributed in distinct regions with clear boundaries. Further analysis employing permutational multivariate analysis of variance (PERMANOVA) demonstrated that R^2^ was equal to 0.9319 and P was equal to 0.0025, which was less than 0.05. This finding indicates a highly significant difference of 93.19% in flavor characteristics among pigments derived from different sources of jujube. The results of Loadings analysis [Fig. 2(C)] demonstrate that the differences in the taste of jujube pigments were mainly manifested in the electronic tongue sensors that are sensitive to astringency, aftertaste-B, umami, richness, etc. This finding suggests that the electronic tongue, when integrated with PCA and Loadings analysis, can effectively discriminate the variations in the taste of jujube pigments from different sources.

### 3.3 Identification of pigment components in jujubes using UPLC-Q-MS/MS

Utilizing the UPLC-Q-TOF-MS/MS technology, the non - volatile chemical substance groups of jujube pigment samples from different sources, namely BJP, DJP, and FJP, were systematically characterized in positive and negative ion modes respectively. The mass spectrometry information of each chemical component was presented in Table 2, Table 3, Table 4, Table 5, Table 6, Table 7. In positive ion mode, 22, 24, and 23 non-volatile compounds were preliminarily identified in BJP, DJP, and FJP, respectively. These compounds included flavonoids (10, 11, and 10), phenolic acids (5), anthraquinones (3), and phenols (4). In the negative ion mode, 23, 20, and 24 compounds were identified, which were classified as flavonoids (11, 9, and 12), phenolic acids (5), anthraquinones (3), and phenols (4, 3, and 4). In this study, a total of 13 kind of flavonoids, 3 anthraquinone compounds, 5 phenolic acids, and 4 phenols were identified from the jujube pigment. Under positive and negative ion modes, a total of 25 non - volatile compounds were identified. Flavonoids accounted for the largest proportion in terms of both types and peak areas.

**Table 2:** The results of component identification of BJP in the positive ion mode.

t/min	Proposed phytochemicals	Classification	Formula	Adduct	Peak	ES Theoretical *m*/*z*	ES found m/z	Mass fragments	δ/ppm
1.105	Rosmarinic acid	Phenols acid	C_18_H_16_O_8_	[M + H]^+^	1,461,231	361.09234	361.0899	358.1647/383.0735/399.0474	8.29
1.189	Epicatechin	Flavonoid	C_15_H_14_O_6_	[M + H]^+^	295,968	291.08686	313.068	286.1439/291.0850/313.0680/329.0438	−6.44
1.189	Quercetin 3-O-neohesperidoside	Flavonoid	C_27_H_30_O_16_	[M + H]^+^	615,848	611.16121	611.1583	609.4723/611.1605	−2.76
1.189	Rhein	Anthraquinones	C_15_H_8_O_6_	[M + H]^+^	1,018,853	285.03991	307.0211	285.0371/307.0222/321.0586	−7.12
1.189	Physcion	Anthraquinones	C_16_H_12_O_5_	[M + H]^+^	118,761	285.07630	285.0736	285.0736/307.0608/321.0588	4.74
1.189	Quercetin 7-O-(6”-O-malonyl)-beta-D-glucoside	Flavonoid	C_24_H_22_O_15_	[M + H]^+^	2,678,471	551.10370	573.0854	551.0992/569.2645/589.0580	0.88
1.205	7,3′,4′-Trihydroxyflavone	Flavonoid	C_15_H_10_O_5_	[M + H]^+^	1,256,952	271.06065	271.0579	291.0478/309.0149	5.05
1.205	Kaempferol	Flavonoid	C_15_H_10_O_6_	[M + H]^+^	757,291	287.05556	309.0376	287.0528/309.0375/321.0586/325.0099	−6.07
1.509	Galbinic Acid	Phenols acid	C_20_H_14_O_11_	[M + H]^+^	1,759,799	431.06144	453.0426	431.0587/448.1967/453.0440	−4.32
1.763	Quercitrin	Flavonoid	C_21_H_20_O_11_	[M + H]^+^	3,805,981	449.10839	471.0902	448.1966/471.0900/487.0657	−2.03
2.878	Syringic acid	Phenols acid	C_9_H_10_O_5_	[M + H]^+^	597,607	199.06065	221.0423	199.0588/221.0425/237.0156	−4.35
2.929	Trans- 4 - Coumaric acid	Phenols acid	C_9_H_8_O_3_	[M + H]^+^	3,477,415	165.05517	165.0535	165.0547/187.0365/203.0109	−6.53
4.145	Procyanidin B3	Phenols	C_30_H_26_O_12_	[M + H]^+^	1,792,744	579.15025	579.1479	579.1484/601.1309/613.1947/617.1103	−2.57
5.564	Procyanidin B-5 3,3′-di-O-gallate	Phenols	C_44_H_34_O_20_	[M + H]^+^	262,915	883.17217	883.1709	883.1709/905.1484/923.1417	2.26
5.869	Chrysin	Flavonoid	C_15_H_10_O_4_	[M + H]^+^	71,225	255.06573	277.0467	257.1361/277.0482/293.0232	−1.27
6.681	Quercetin 3-O-rutinoside-(1- > 2)-O-rhamnoside [Quercetin-3-O-(2G-alpha-L-rhamnosyl)-rutinoside]	Flavonoid	C_33_H_40_O_20_	[M + H]^+^	56,928	757.21912	757.2184	757.2184/779.1997/795.1724	−1.4
7.644	Caffeic acid	Phenols acid	C_9_H_8_O_4_	[M + H]^+^	166,564	181.05008	181.0494	181.0495/203.0326/219.0026	−0.72
8.05	Resveratrol	Phenols	C_14_H_12_O_3_	[M + H]^+^	150,390	229.08647	229.0863	225.1098/229.0867/251.0749/267.0459	1.66
8.556	2,3,5,4’-Tetrahydroxyl diphenylethylene-2-O-glucoside	Phenols	C_20_H_22_O_9_	[M + H]^+^	35,200	407.13421	407.1314	407.1315/429.1147/435.0902/445.0885	−2.07
8.556	5-Hydroxy-3′,4′,7-trimethoxyflavone	Flavonoid	C_18_H_16_O_6_	[M + H]^+^	2,272,898	329.10251	351.0838	329.0995/351.0838/367.0603	−0.55
9.875	Emodinanthrone	Anthraquinones	C_15_H_12_O_4_	[M + H]^+^	273,728	257.08138	257.0781	257.0781/279.0606/295.0355	−9.45
10.736	Keracyanin	Flavonoid	C_27_H_31_O_15_	[M + H]^+^	99,073	595.16630	596.1699	595.1650/618.1482/634.1287	−5.6

**Table 3:** The results of component identification of BJP in the negative ion mode.

t/min	Proposed phytochemicals	Classification	Formula	Adduct	Peak	ES Theoretical m/z	ES found m/z	Mass fragments	δ/ppm
1.162	Galbinic Acid	Phenols acid	C_20_H_14_O_11_	[M-H]^−^	2,669,498	429.04579	429.0442	429.0452/433.1137/475.0503	−3.91
1.179	Rosmarinic acid	Phenols acid	C_18_H_16_O_8_	[M-H]^−^	50,181	359.07669	359.077	355.0667/359.0774/405.0823	−0.04
1.179	Epicatechin	Flavonoid	C15H14O6	[M-H]^−^	251,041	290.07904	335.0788	299.0732/291.0122/335.0787	5.58
1.197	Keracyanin	Flavonoid	C_27_H_31_O_15_	[M-H]^−^	53,721	594.15847	594.1673	589.1985/594.1673/640.1488	−4.21
1.365	Kaempferol	Flavonoid	C_15_H_10_O_6_	[M + HCOO]^−^	269,316	285.03991	285.0406	285.0405/331.0457	0.65
1.619	7,3′,4′-Trihydroxyflavone	Flavonoid	C_15_H_10_O_5_	[M-H]^−^	2,146,937	269.04500	315.0518	269.0461/272.0754/315.0508	1
1.922	Chrysin	Flavonoid	C_15_H_10_O_4_	[M + HCOO]^−^	573,968	253.05008	299.0563	299.0562/300.0598/301.0613	1.14
2.885	Quercitrin	Flavonoid	C_21_H_20_O_11_	[M-H]^−^	3,629,690	447.09274	447.0925	443.1401/447.0927/493.0977	−1.73
3.646	Physcion	Anthraquinones	C_16_H_12_O_5_	[M + HCOO]^−^	810,226	283.06065	283.0599	284.0388/329.0630	−4.13
3.899	Syringic acid	Phenols acid	C_9_H_10_O_5_	[M + HCOO]^−^	528,776	197.04500	197.0455	197.0456/243.0509	−1.68
5.368	2,3,5,4’-Tetrahydroxyl diphenylethylene-2-O-glucoside	Phenols	C_20_H_22_O_9_	[M-H]^−^	3,346,545	406.12638	451.1238	405.1205/151.1245/453.0655	−1.29
5.622	Trans- 4 - Coumaric acid	Phenols acid	C_9_H_8_O_3_	[M-H]^−^	55,160	163.03952	209.0454	159.0664/163.0401/209.0456	−0.58
6.249	Quercetin 3-O-rutinoside-(1- > 2)-O-rhamnoside [Quercetin-3-O-(2G-alpha-L-rhamnosyl)-rutinoside]	Flavonoid	C_33_H_40_O_20_	[M + HCOO]^−^	83,523	755.20347	755.2046	755.2029/801.2240	−3.01
6.384	Procyanidin B3	Phenols	C_30_H_26_O_12_	[M-H]^−^	17,852	577.13460	623.1421	573.1238/577.1361/623.1374	1.17
7.093	Caffeic acid	Phenols acid	C_9_H_8_O_4_	[M-H]^−^	902,016	179.03443	179.0351	179.0351/180.0384/181.0396	0.62
9.376	Procyanidin B-5 3,3′-di-O-gallate	Phenols	C_44_H_34_O_20_	[M-H]^−^	464,449	881.15652	881.1567	881.1607/882.1511/887.1470	−0.41
9.577	Quercetin 3-O-neohesperidoside	Flavonoid	C_27_H_30_O_16_	[M-H]^−^	3,369,646	609.14556	609.1464	605.1223/609.1451/610.1523	0.42
9.628	4′,6,7-Trimethoxyisoflavone	Flavonoid	C_18_H_16_O_5_	[M-H]^−^	960,005	311.09195	357.0981	311.0935/359.2077	0.41
9.729	5-Hydroxy-3′,4′,7-trimethoxyflavone	Flavonoid	C_18_H_16_O_6_	[M-H]^- -^	5,506,312	327.08686	327.0877	327.0876/373.0927	0.86
11.676	Rhein	Anthraquinones	C_15_H_8_O_6_	[M + HCOO]^−^	212,215	283.02426	329.0306	327.2195/329.0304/331.0376	1.51
11.727	Resveratrol	Phenols	C_14_H_12_O_3_	[M + HCOO]^−^	149,528	227.07082	273.0783	272.0754/273.0782/274.0786/275.0865	5.68
16.223	Quercetin 7-O-(6”-O-malonyl)-beta-D-glucoside	Flavonoid	C_24_H_22_O_15_	[M-H]^−^	29,041	549.08804	549.0872	549.0880/590.2020/595.0943	−5.88
19.824	Emodinanthrone	Anthraquinones	C_15_H_12_O_4_	[M + HCOO]^−^	38,085	255.06573	301.0724	301.1290/302.0768	2.83

**Table 4:** The results of component identification of DJP in the positive ion mode.

t/min	Proposed phytochemicals	Classification	Formula	Adduct	Peak	ES Theoretical m/z	ES found m/z	Mass fragments	δ/ppm
1.168	Rosmarinic acid	Flavonoid	C_18_H_16_O_8_	[M + H]^+^	4,583,096	361.09234	383.0736	361.0894/381.0799/399.0490	−2.3
1.168	Kaempferol	Flavonoid	C_15_H_10_O_6_	[M + H]^+^	3,768,748	287.05556	309.0371	287.0528/291.0478/309.0371/325.0087	−6.4
1.184	7,3′,4′-Trihydroxyflavone	Flavonoid	C_15_H_10_O_5_	[M + H]^+^	526,853	271.06065	271.0579	268.0795/293.0425/309.0158	−4.13
1.184	2,3,5,4’-Tetrahydroxyl diphenylethylene-2-O-glucoside	Phenols	C_20_H_22_O_9_	[M + H]^+^	73,242	407.13421	429.1159	407.1321/429.1158/448.1966	3.23
1.184	Quercetin 3-O-neohesperidoside	Flavonoid	C_27_H_30_O_16_	[M + H]^+^	27,914	611.16121	611.1615	611.1600/633.1427	−1.22
1.201	Rhein	Anthraquinones	C_15_H_8_O_6_	[M + H]^+^	872,949	285.03991	307.0209	287.0528/307.0209/322.9927	−7.38
1.218	Emodinanthrone	Anthraquinones	C_15_H_12_O_4_	[M + H]^+^	77,938	257.08138	279.066	255.1208/279.0611/295.0451	9.53
1.471	Galbinic Acid	Phenols acid	C_20_H_14_O_11_	[M + H]^+^	1,354,922	431.06144	453.042	431.0586/455.1527	−5.32
1.522	Quercetin 7-O-(6”-O-malonyl)-beta-D-glucoside	Flavonoid	C_24_H_22_O_15_	[M + H]^+^	3,460,986	551.10370	573.0864	552.1697/573.0827/589.0551	2.21
1.573	Epicatechin	Flavonoid	C_15_H_14_O_6_	[M + H]^+^	1,449,256	291.08686	291.0841	289.0684/313.0684/329.0455	−7.47
1.674	Physcion	Anthraquinones	C_16_H_12_O_5_	[M + H]^+^	3,872,375	285.07630	307.0585	284.0531/307.0580	−3.99
1.725	Chrysin	Flavonoid	C_15_H_10_O_4_	[M + H]^+^	2,115,911	255.06573	277.0469	257.0637/277.0499/293.0223	−6.22
2.738	Quercitrin	Flavonoid	C_21_H_20_O_11_	[M + H]^+^	5,102,493	449.10839	449.1055	449.1046/453.1003/471.0899/487.0626	−4.57
3.347	Keracyanin	Flavonoid	C_27_H_31_O_15_	[M + H]^+^	1,781,486	596.17412	634.1323	595.1639/634.1320	−8.55
3.904	Syringic acid	Phenols acid	C_9_H_10_O_5_	[M + H]^+^	868,739	199.06065	199.0596	194.0789/199.0587/221.0425/237.0144	−2.7
5.374	Trans- 4 - Coumaric acid	Phenols acid	C_9_H_8_O_3_	[M + H]^+^	336,354	165.05517	165.0532	165.0546/187.0366/203.0116	−8.6
5.881	Procyanidin B3	Phenols	C_30_H_26_O_12_	[M + H]^+^	4,392,912	579.15025	601.1338	575.1736/579.1462/601.1338/617.1013	−2.45
6.033	Quercetin 3-O-rutinoside-(1- > 2)-O-rhamnoside [Quercetin-3-O-(2G-alpha-L-rhamnosyl)-rutinoside]	Flavonoid	C_33_H_40_O_20_	[M + H]^+^	6,971,890	757.21912	779.1981	327.2145/351.0836/367.0577	−3.18
7.097	Procyanidin B-5 3,3′-di-O-gallate	Phenols	C_44_H_34_O_20_	[M + H]^+^	180,676	883.17217	905.1577	883.1698/922.0096	−0.96
7.15	Caffeic acid	Phenols acid	C_9_H_8_O_4_	[M + H]^+^	140,657	181.05008	181.0494	181.0477/203.0317/214.0479/219.0091	−0.99
8.62	5-Hydroxy-3′,4′,7-trimethoxyflavone	Flavonoid	C_18_H_16_O_6_	[M + H]^+^	718,441	329.10251	351.0836	329.0999/351.0836/367.0582	−1.12
8.721	Beta-Phenylpropiophenone	Flavonoid	C_15_H_14_O	[M + H]^+^	708,965	211.11229	211.1076	213.0909	−7.31
10.495	Resveratrol	Phenols	C_14_H_12_O_3_	[M + H]^+^	702,598	229.08647	229.0838	229.0838/251.0719	−9.19

**Table 5:** The results of component identification of DJP in the negative ion mode.

t/min	Proposed phytochemicals	Classification	Formula	Adduct	Peak	ES Theoretical m/z	ES found m/z	Mass fragments	δ/ppm
1.162	Galbinic Acid	Phenols acid	C_20_H_14_O_11_	[M + HCOO]^−^	2,121,109	429.04579	429.0438	431.1406/475.0498	−5.42
1.179	Rosmarinic acid	Phenols acid	C_18_H_16_O_8_	[M-H]^−^	4,588,222	359.07669	405.0815	355.1034/359.0775/405.0820	−3.75
1.179	Epicatechin	Flavonoid	C15H14O6	[M + HCOO]^−^	203,650	290.07904	289.0705	287.0775/335.0781	−8.74
1.365	Kaempferol	Flavonoid	C_15_H_10_O_6_	[M + HCOO]^−^	376,170	285.03991	331.0443	331.0443/333.0232	−6.58
1.619	7,3′,4′-Trihydroxyflavone	Flavonoid	C_15_H_10_O_5_	[M + HCOO]^−^	268,146	269.04500	315.0525	269.0667/315.0525	−7.17
1.922	Chrysin	Flavonoid	C_15_H_10_O_4_	[M + HCOO]^−^	172,520	253.05008	299.055	299.0560/302.0858	−4.03
2.885	Quercitrin	Flavonoid	C_21_H_20_O_11_	[M + HCOO]^−^	3,059,868	447.09274	447.0922	443.1188/493.0973	−3.7
3.646	Physcion	Anthraquinones	C_16_H_12_O_5_	[M + HCOO]^−^	1,720,585	283.06065	283.059	283.0825/329.0667	0.11
3.899	Syringic acid	Phenols acid	C_9_H_10_O_5_	[M-H]^−^	5,410,178	197.04500	197.0454	197.0455/203.0564/243.0511	−0.26
5.368	2,3,5,4’-Tetrahydroxyl diphenylethylene-2-O-glucoside	Phenols	C_20_H_22_O_9_	[M + HCOO]^−^	7,219,302	406.12638	405.119	409.1138/154.1242	−1.61
5.622	Trans- 4 - Coumaric acid	Phenols acid	C_9_H_8_O_3_	[M-H]^−^	561,528	163.03952	209.0456	163.0401/213.0042	0.48
6.384	Procyanidin B3	Phenol	C_30_H_26_O_12_	[M + HCOO]^−^	30,192	577.13460	623.1349	623.1349/625.1528/628.1756	−5.98
7.093	Caffeic acid	Phenols acid	C_9_H_8_O_4_	[M + HCOO]^−^	1,925,555	179.03443	179.0351	179.0351/225.0403	0.85
9.577	Quercetin 3-O-neohesperidoside	Flavonoid	C_27_H_30_O_16_	[M-H]^−^	152,211	609.14556	609.1462	609.1459/610.1506	−0.04
9.628	4′,6,7-Trimethoxyisoflavone	Flavonoid	C_18_H_16_O_5_	[M + HCOO]^−^	339,589	311.09195	357.0978	357.0980	−0.16
9.729	5-Hydroxy-3′,4′,7-trimethoxyflavone	Flavonoid	C_18_H_16_O_6_	[M + HCOO]^−^	914,485	327.08686	327.0875	327.0875/373.0924	0.13
11.676	Rhein	Anthraquinones	C_15_H_8_O_6_	[M-H]^−^	127,024	283.02426	329.0293	283.0249/287.1502/329.0289	−4.07
11.727	Resveratrol	Phenols	C_14_H_12_O_3_	[M + HCOO]^−^	58,493	227.07082	273.0775	273.0771/277.0674	3.89
16.223	Quercetin 7-O-(6”-O-malonyl)-beta-D-glucoside	Flavonoid	C_24_H_22_O_15_	[M + HCOO]^−^	45,421	549.08804	549.0882	547.1448/595.0977	−7.2
19.824	Emodinanthrone	Anthraquinones	C_15_H_12_O_4_	[M-H]^−^	119,056	255.06573	255.0664	255.0664/301.0734/307.0441	1.17

**Table 6:** The results of component identification of FJP in the positive ion mode.

t/min	Proposed phytochemicals	Classification	Formula	Adduct	Peak	ES Theoretical m/z	ES found m/z	Mass fragments	δ/ppm
1.185	Kaempferol	Flavonoid	C_15_H_10_O_6_	[M + H]^+^	2,258,121	287.05556	309.0371	284.0530/309.0370/325.0087	−6.2
1.185	Epicatechin	Flavonoid	C_15_H_14_O_6_	[M + H]^+^	310,547	291.08686	313.0683	291.0840/313.0683/330.0599	−2.86
1.202	7,3′,4′-Trihydroxyflavone	Flavonoid	C_15_H_10_O_5_	[M + H]^+^	259,021	271.06065	271.0579	271.0578/291.0478	−4.24
1.202	Quercetin 3-O-neohesperidoside	Flavonoid	C_27_H_30_O_16_	[M + H]^+^	59,497	611.16121	649.1148	611.1600/633.1435/649.1243	−2.72
1.523	Quercetin 7-O-(6”-O-malonyl)-beta-D-glucoside	Flavonoid	C_24_H_22_O_15_	[M + H]^+^	1,414,413	551.10370	573.0801	551.0983/573.0810/579.0722/589.0547	−8.19
1.675	Physcion	Anthraquinones	C_16_H_12_O_5_	[M + H]^+^	3,160,874	285.07630	307.0582	286.0584/307.0584/323.0371	1.77
1.726	Galbinic Acid	Phenolic acids	C_20_H_14_O_11_	[M + H]^+^	1,418,891	431.06144	431.0587	431.0597/455.1529/469.0131	−4.82
1.776	Rhein	Anthraquinones	C_15_H_8_O_6_	[M + H]^+^	2,011,881	285.03991	285.0369	285.0371/291.0478/307.0214/322.9928	−8.18
1.827	Chrysin	Flavonoid	C_15_H_10_O_4_	[M + H]^+^	2,446,078	255.06573	277.0469	255.0622/277.1257	−6.52
2.03	Emodinanthrone	Anthraquinones	C_15_H_12_O_4_	[M + H]^+^	1,240,076	257.08138	279.0628	257.0783/261.1100/279.0652	−1.1
2.689	2,3,5,4’-Tetrahydroxyl diphenylethylene-2-O-glucoside	Phenols	C_20_H_22_O_9_	[M + H]^+^	752,908	407.13421	407.132	409.1107/429.1158/445.0886	−3.32
2.739	Quercitrin	Flavonoid	C_21_H_20_O_11_	[M + H]^+^	6,020,695	449.10839	471.0896	449.1055/455.1529/471.0905/487.0634	−3.24
3.044	Procyanidin B3	Phenols	C_30_H_26_O_12_	[M + H]^+^	3,837,130	579.15025	601.1313	579.1522/599.1579/617.1035	0.52
3.753	Caffeic acid	Phenolic acids	C_9_H_8_O_4_	[M + H]^+^	319,887	181.05008	181.049	181.0481/198.0529/203.0319/219.0047	−4.32
3.905	Syringic acid	Phenolic acids	C_9_H_10_O_5_	[M + H]^+^	2,411,880	199.06065	199.0596	199.0598/221.0423/237.0742	−1.74
5.375	Trans- 4 - Coumaric acid	Phenolic acids	C_9_H_8_O_3_	[M + H]^+^	719,204	165.05517	165.0541	165.0547/187.0365/203.0107	−3.55
5.882	Rosmarinic acid	Phenolic acids	C_18_H_16_O_8_	[M + H]^+^	364,178	361.09234	361.0896	361.0896/381.1160/399.0474	−5.33
6.695	Quercetin 3-O-rutinoside-(1- > 2)-O-rhamnoside [Quercetin-3-O-(2G-alpha-L-rhamnosyl)-rutinoside]	Flavonoid	C_33_H_40_O_20_	[M + H]^+^	365,682	757.21912	757.2193	757.2144/779.1988	2.27
8.562	Keracyanin	Flavonoid	C_27_H_31_O_15_	[M + H]^+^	152,322	596.17412	596.1769	591.2630/596.1769/618.1610	4.15
8.621	5-Hydroxy-3′,4′,7-trimethoxyflavone	Flavonoid	C_18_H_16_O_6_	[M + H]^+^	656,172	329.10251	351.0838	327.2144/351.0837	−1.06
10.293	Resveratrol	Flavonoid	C_14_H_12_O_3_	[M + H]^+^	149,991	229.08647	229.0835	231.1706/251.0681	−8.91
16.575	Procyanidin B-5 3,3′-di-O-gallate	Phenols	C_44_H_34_O_20_	[M + H]^+^	57,631	883.17217	883.1754	883.1754/905.1404/922.0097	−2.77

**Table 7:** The results of component identification of FJP in the negative ion mode.

t/min	Proposed phytochemicals	Classification	Formula	Adduct	Peak	ES Theoretical m/z	ES found m/z	Mass fragments	δ/ppm
1.184	Epicatechin	Flavonoid	C_15_H_14_O_6_	[M-H]^−^/[M + HCOO^]-^	106,831	289.07121	335.078	289.0716/293.1032/335.0770	3.08
1.184	Quercetin 3-O-neohesperidoside	Flavonoid	C_27_H_30_O_16_	[M-H]^−^	63,062	609.14556	609.1446	609.1454/653.2145	−4.31
1.471	Quercetin 7-O-(6”-O-malonyl)-beta-D-glucoside	Flavonoid	C_24_H_22_O_15_	[M-H]^−^/[M + HCOO]^−^	849,177	549.08804	549.0864	545.1723/549.0875/595.0907	−5.39
1.623	Procyanidin B-5 3,3′-di-O-gallate	Phenols	C_44_H_34_O_20_	[M-H]^−^	831,837	881.15652	881.158	881.1574/925.1859	−0.25
1.674	Galbinic Acid	Phenols acid	C_20_H_14_O_11_	[M + HCOO]^−^	1,468,618	429.04579	475.0508	431.1412/475.0499	−2.94
2.13	Quercetin 3-O-rutinoside-(1- > 2)-O-rhamnoside [Quercetin-3-O-(2G-alpha-L-rhamnosyl)-rutinoside]	Flavonoid	C_33_H_40_O_20_	[M-H]−/[M + HCOO]^−^	51,181	755.20347	755.204	755.2019/801.1979/807.1662	−0.05
2.475	Chrysin	Flavonoid	C_15_H_10_O_4_	[M + HCOO]^−^	63,310	253.05008	253.0479	255.0510/299.0555	−6.76
2.687	Quercitrin	Flavonoid	C_21_H_20_O_11_	[M-H]^−^	6,510,066	447.09274	447.0923	447.0921/491.1620	−3.66
2.687	Rosmarinic acid	Phenols acid	C_18_H_16_O_8_	[M + HCOO]^−^	229,480	359.07669	405.0815	363.1085/405.0822	−3.51
3.944	BETA-PHENYLPROPIOPHENONE	Flavonoid	C_15_H_14_O	[M + HCOO]^−^	77,338	209.09664	623.1349	255.0876/256.1049	−9.81
3.955	Syringic acid	Phenols acid	C_9_H_10_O_5_	[M-H]^−^/[M + HCOO]^−^	6,291,091	197.04500	243.0511	197.0455/203.0563/243.0512	−0.04
5.323	2,3,5,4’-Tetrahydroxyl diphenylethylene-2-O-glucoside	Phenols	C_20_H_22_O_9_	[M-H]^−^	383,001	405.11856	405.1192	405.1190/448.1826	−0.77
5.425	Trans- 4 - Coumaric acid	Phenols acid	C_9_H_8_O_3_	[M-H]^−^	532,480	165.05517	163.0401	163.0402/165.0194	0.78
6.176	Keracyanin	Flavonoid	C_27_H_31_O_15_	[M + HCOO]^−^	224,044	594.15847	640.1651	593.2450/640.1651	−5.27
6.286	7,3′,4′-Trihydroxyflavone	Flavonoid	C_15_H_10_O_5_	[M + HCOO]^−^	134,109	269.04500	315.0501	311.1138/315.0504	−1.99
7.15	Caffeic acid	Phenols acid	C_9_H_8_O_4_	[M-H]^−^/[M + HCOO]^−^	2,813,635	179.03443	179.0351	179.0351/185.0457/225.0396	0.79
8.609	Procyanidin B3	Phenols	C_30_H_26_O_12_	[M-H]^−^	77,338	577.13460	577.1205	577.1205/625.1849	−3.88
8.62	5-Hydroxy-3′,4′,7-trimethoxyflavone	Flavonoid	C_18_H_16_O_6_	[M-H]^−^/[M + HCOO]^−^	10,508,448	327.08686	327.0878	327.0875/373.0924	1.03
8.67	Physcion	Anthraquinones	C_16_H_12_O_5_	[M + HCOO]^−^	96,820	283.06065	329.0657	327.0878/329.0657	−3.28
9.633	4′,6,7-Trimethoxyisoflavone	Flavonoid	C_18_H_16_O_5_	[M-H]^−^	385,228	311.09195	357.098	311.0928/357.0981	−0.88
13.507	Resveratrol	Phenols	C_14_H_12_O_3_	[M + HCOO]^−^	56,829	227.07082	273.0771	269.0668/273.0782	1.12
16.703	Kaempferol	Flavonoid	C_15_H_10_O_6_	[M-H]^−^	39,162	285.03991	285.0404	285.0407/286.0433	−0.2
16.703	Rhein	Anthraquinones	C_15_H_8_O_6_	[M + HCOO]^−^	58,233	283.02426	329.0299	327.2186/329.0304	−0.19
19.745	Emodinanthrone	Anthraquinones	C_15_H_12_O_4_	[M-H]^−^/[M + HCOO]^−^	31,710	255.06573	255.0661	255.0619/259.0824/301.0733	0.14

Peak area comparison: In the pigment components of BJP, DJP, and FJP jujubes, 22 and 19 common compounds were detected under positive and negative ion modes, respectively. The relative change rates of each compound were calculated according to their average peak areas. The detailed results are presented in Table 1, Table 2 of the Supporting Information. It is noteworthy that the variation rates of these common compounds among BJP, DJP, and FJP did not exhibit any distinct patterns.

### 3.4 PLS-DA analysis of jujube pigment components

**Fig. 3:**
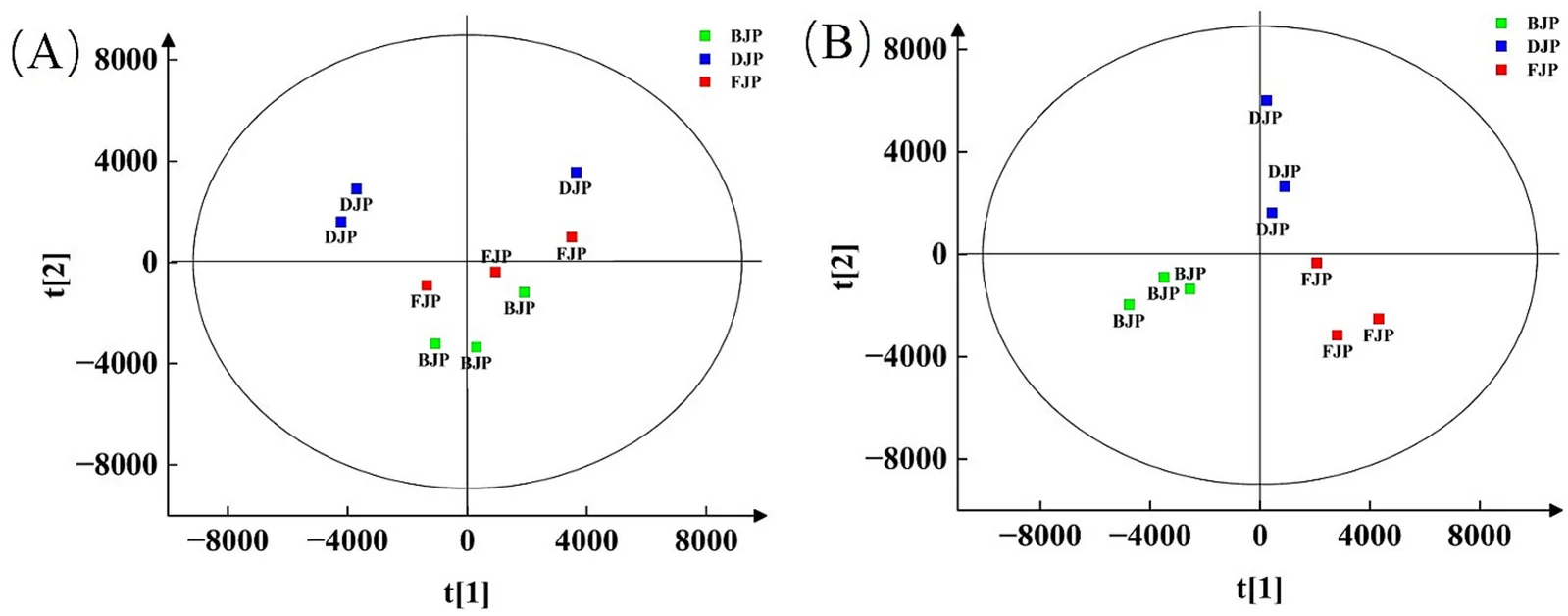
PCA analysis of jujube pigment from different sources. (A) Positive ion, (B) Negative ion.

Principal component analysis is capable of achieving effective dimensionality reduction and comprehensively characterizing non-volatile components. The mass-to-charge ratio, retention time, peak area, and other relevant data of BJP, DJP, and FJP were imported into the Sieve v2.1 (×64) software. Subsequently, the processed data were imported into the SIMCA 13.0 software for PLS - DA multivariate analysis. The PCA results are shown in Fig. 3(A) and (B). In the positive ion mode, the contribution rates of PC1 and PC2 were 30.7% and 23.0%, respectively. In the negative ion mode, the contribution rates of PC1 and PC2 are 31.4% and 29.4%, respectively, accounting for 53.7% and 60.8% of the sample information volume, respectively.

**Fig. 4:**
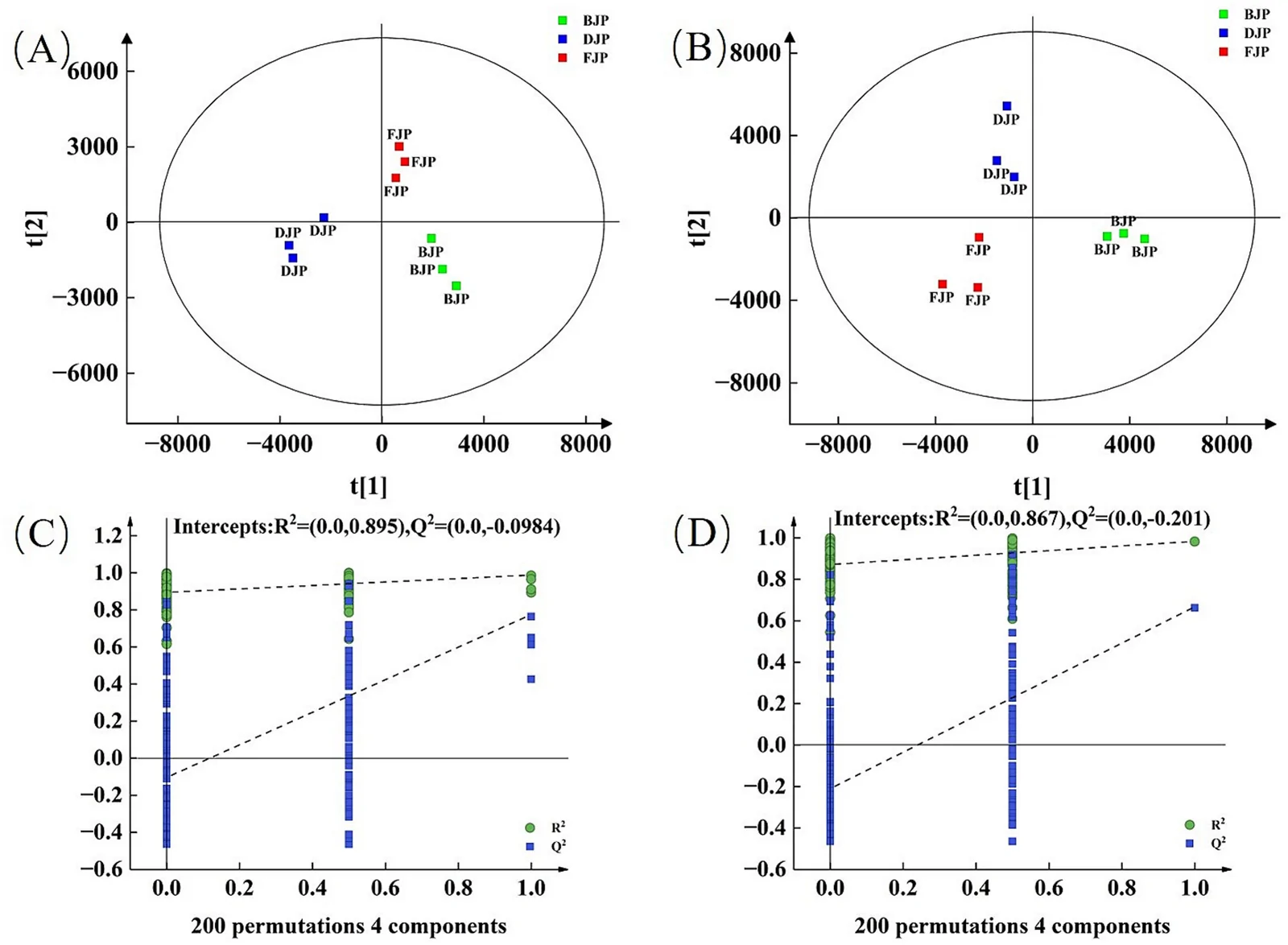
PLS-DA and permutation test analysis of jujube pigment from different sources. (A) and (C) Positive ion, (B) and (D) Negative ion.

In the positive and negative ion modes, the BJP, DJP, and FJP samples were discretely distributed on the two – dimensional (2D) plane of the PLS - DA model, as shown in Fig. 4 (A) and (B). Furthermore, these three samples could be clearly distinguished. For the positive ion model parameters, the fitting index R2X of the independent variable was 0.752, the fitting index R2Y of the dependent variable was 0.99, and the model prediction index Q2 was 0.83. For the negative ion model parameters, the fitting index R2X of the independent variable was 0.777, the fitting index R2Y of the dependent variable was 0.982, and the model prediction index Q2 was 0.779. The R2 and Q2 values of both the positive and negative ion models were greater than 0.5, indicating that the model fitting effect met the acceptable standard (Yun et al., 2021). Fig. 4(C) and 4(D) display the results of 200 permutation test analyses conducted by the PLS - DA model. All the square and circular points have their x - coordinates located to the left of the original values on the right - hand side. The intercept of the Q2 regression line with the Y - axis is less than 0, indicating that there is no overfitting in the model. The validation results of the model are reliable, and thus, it can be inferred that the non - volatile substances of the pigments of the three types of red dates, namely BJP, DJP, and FJP, can all be effectively differentiated.

### 3.5 Analysis of key differential components in jujube pigments from different sources

The variations in the pigment difference components of jujubes from diverse sources (refer to Table 1, Table 2 in the supporting documents) were determined using variable importance in projection (VIP) values of positive and negative ion modes variables. As the VIP value rises, the influence of the change in the proportion of compound composition within the data becomes more pronounced. Based on the screening criterion of VIP value >1, a total of 13 key differential components were identified in both positive and negative ion modes, and these components are presented in Table 8.

**Table 8:** Key differential compounds of jujube pigment in positive and negative ion modes.

Number	Positive ion mode			Negative ion mode		
	Proposed phytochemicals	VIP	p	Proposed phytochemicals	VIP	p
1	Rosmarinic acid	2.16673	0.066	Rosmarinic acid	1.07666	0.361
2	Physcion	1.67806	0.051	–		
3	2,3,5,4’-Tetrahydroxyl diphenylethylene-2-O-glucoside	1.52449	0.393	2,3,5,4’-Tetrahydroxyl diphenylethylene-2-O-glucoside	2.33092	0.066
4	Quercitrin	1.2138	0.148	Quercitrin	1.42227	0.067
5	Quercetin 7-O-(6”-O-malonyl)-beta-D-glucoside	1.21052	0.202	Quercetin 7-O-(6”-O-malonyl)-beta-D-glucoside	1.21298	0.142
6	Trans- 4 - Coumaric acid	1.19819	0.061	–		
7	Kaempferol	1.19093	0.193	–		
8	Procyanidin B3	1.15505	0.790	–		
9	Quercetin 3-O-rutinoside-(1- > 2)-O-rhamnoside [Quercetin-3-O-(2G-alpha-L-rhamnosyl)-rutinoside]	1.24287	0.871	–		
10	–			Syringic acid	1.98267	0.027
11	–			5-Hydroxy-3′,4′,7-trimethoxyflavone	1.66554	0.156
12	–			Caffeic acid	1.0357	0.004
13	–			Quercetin 3-O-neohesperidoside	1.10897	0.097

Note:“-” not detected.

### 3.6 Correlation between jujube pigment taste and key differential compounds

**Fig. 5:**
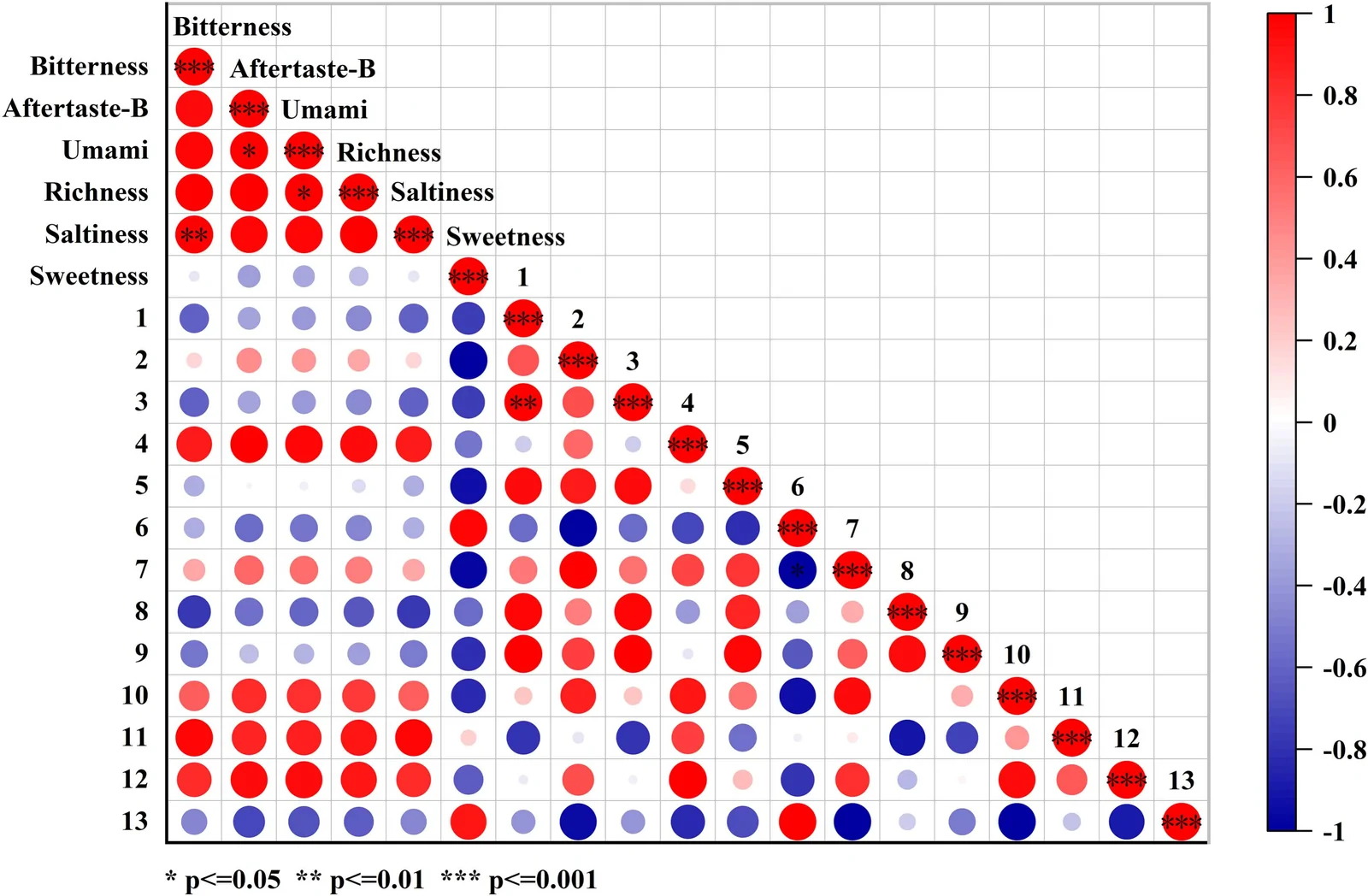
Association analysis between the pigment flavor of jujubes and the key identified compounds.

The exploratory correlation screening between jujube pigment flavor and key compounds is presented in Fig. 5. The bitterness, aftertaste - B, umami, richness, and saltiness of jujube pigment demonstrate a priority ranking relationship among candidate markers, such as caffeic acid, 5-hydroxy-3′, 4′, 7-trimethoxyflavone, syringic acid, and quercetin. This is consistent with the higher response value of the electronic tongue for bitter taste as depicted in Fig. 2. Its sweetness also demonstrates a priority ranking of candidate biomarkers along with trans-4-coumaric acid and quercetin 3-O-neohesperidin.

## 4 Conclusion

Natural pigments are classified into anthocyanins, flavonoids, quinones, tetrapyrroles, and carotenoids based on their chemical structures. This experimental study revealed that the pigment of jujube is soluble in water, and visually, the color of its solution does not undergo significant changes with the variation of pH. Besides the initial detection of suspected anthraquinone substances in jujube pigment, flavonoids, phenols, and phenolic acid components were also detected. These components are similar to the composition of polyphenols and phenolic compounds in jujubes reported by Aneta et al. (2016) and Dou et al. (2023), suggesting that jujube pigment is a type of mixed natural pigment. BJP exhibits better water - solubility than FJP and DJP. This might be because the water - soluble components such as sugars and proteins in the jujube peel have been mostly dissolved during the processing of jujubes. The peak areas of the identified compounds in the three sample pigments were compared, and no obvious regular patterns were found. Moreover, the taste characteristics of the three pigments were highly similar. Umami, saltiness, aftertaste, Aftertaste - B, and sweetness were the key taste characteristics of jujube pigments. The flavor characteristics of jujube pigment, such as bitterness, sweet aftertaste (B), umami, mellow taste, and saltiness, are positively correlated with caffeic acid, 5 - hydroxy - 3′,4′, 7 - trimethoxyethanol, eugenic acid, and quercetin glycoside. The sweetness is positively correlated with trans - 4 - coumaric acid and quercetin 7 - O - (6″ - O - malonyl) - β - D - glucoside.

This study utilized an association analysis approach that integrated an electronic tongue and non - targeted mass spectrometry. In essence, this approach falls within a high - throughput screening framework, with the objective of identifying chemical markers that exhibit a covariant relationship with taste responses from complex matrices. Considering that there is no publicly accessible taste threshold data for certain differential compounds, the taste activity value (TAV) was not computed in this study. Consequently, the correlation presented in Fig. 5 should be interpreted as the “priority ranking of candidate markers” rather than the “flavor contribution theory”. Future research will conduct absolute quantification and sensory recombination verification on the key compounds that have been screened out, thus further validating their actual flavor roles. Moreover, owing to the absence of reference standards for the target monomers, the identified compounds cannot exclude the possibility of the existence of isomers. Subsequently, a preliminary confirmation can be achieved by separating, purifying, and preparing the monomer standard substances. Further experimental research and verification are still required regarding the degradation or transformation of phenolic and flavonoid substances during the extraction process using high - concentration alkaline solvents.

## CRediT authorship contribution statement

**Junlan Li:** Writing – original draft, Supervision, Methodology, Investigation, Funding acquisition, Data curation. **Shunmeng Chen:** Resources, Formal analysis, Data curation, Conceptualization. **Yuchun Zhou:** Resources, Formal analysis, Data curation.

## Declaration of competing interest

The authors declare the following financial interests/personal relationships which may be considered as potential competing interests: Junlan Li reports financial support was provided by the Industry Support Program of the Gansu Provincial Department of Education. Reports a relationship with that includes:. Has patent pending to. If there are other authors, they declare that they have no known competing financial interests or personal relationships that could have appeared to influence the work reported in this paper.

## Data Availability

No data was used for the research described in the article.
