# Supplementary material for: Analysis of taste and non-volatile compound differences in jujube pigments from different sources using multiple techniques
Source: Food Chem X. 2026 Aug 28;39:104378. doi: 10.1016/j.fochx.2026.104378 (PMC13571703; doi:10.1016/j.fochx.2026.104378)
Supplement: Supplementary file 1 [file mmc1.docx]

**Analysis of** **taste and non-volatile compound differences in jujube pigments from different sources using multiple techniques**

Junlan Li^[1]^*^,1,2^, Shunmeng Chen^2^, Yuchun Zhou ^3^

1. Institute of Rural Revitalization, Hexi University, Zhangye, Gansu 734000, PR China

2. College of Agriculture and Ecological Engineering, Hexi University, Zhangye, Gansu 734000, China

3. Jiangsu Food & Pharmaceutical Science College, Huaian, Jiangsu 223300, China

Table 1 Comparison of peak areas and analysis of variable importance projection (VIP) values for BJP, DJP, and FJP compounds in positive ion mode

| Proposed phytochemicals | Adduct | Peak | | | VIP | p | Rate of change compared to the average (%) | | |
| --- | --- | --- | --- | --- | --- | --- | --- | --- | --- |
|  |  | BJP | DJP | FJP |  |  | BJP | DJP | FJP |
| Rosmarinic acid | [M+H]^+^ | 1461231 | 4583096 | 364178 | 1.91581 | 0.066 | -31.59 | 114.55 | -82.95 |
| Epicatechin | [M+H]^+^ | 295968 | 1449256 | 310547 | 0.643025 | 0.066 | -56.81 | 111.49 | -54.68 |
| Quercetin 3-O-neohesperidoside | [M+H]^+^ | 615848 | 27914 | 59497 | 0.539315 | 0.061 | 162.71 | -88.09 | -74.61 |
| Rhein | [M+H]^+^ | 1018853 | 872949 | 2011881 | 0.686247 | 0.561 | -21.70 | -32.91 | 54.61 |
| Physcion | [M+H]^+^ | 118761 | 3872375 | 3160874 | 1.49562 | 0.051 | -95.02 | 62.43 | 32.58 |
| Quercetin 7-O-(6''-O-malonyl)-beta-D-glucoside | [M+H]^+^ | 2678471 | 3460986 | 1414413 | 1.11285 | 0.202 | 6.38 | 37.45217 | -43.83 |
| 7,3',4'-Trihydroxyflavone | [M+H]^+^ | 1256952 | 526853 | 259021 | 0.806888 | 0.252 | 84.59 | -22.6288 | -61.96 |
| Kaempferol | [M+H]^+^ | 757291 | 3768748 | 2258121 | 1.09799 | 0.193 | -66.51 | 66.66 | -0.14 |
| Galbinic Acid | [M+H]^+^ | 1759799 | 1354922 | 1418891 | 0.630469 | 0.062 | 16.45 | -10.34 | -6.11 |
| Quercitrin | [M+H]^+^ | 3805981 | 5102493 | 6510066 | 1.20412 | 0.148 | -25.95 | -0.72 | 26.67 |
| Syringic acid | [M+H]^+^ | 597607 | 868739 | 2411880 | 0.681119 | 0.845 | -53.77 | -32.79 | 86.57 |
| trans- 4 - Coumaric acid | [M+H]^+^ | 3477415 | 336354 | 719204 | 1.11116 | 0.061 | 130.14 | -77.73 | -52.40 |
| Procyanidin B3 | [M+H]^+^ | 1792744 | 4392912 | 3837130 | 1.39842 | 0.790 | -46.34 | 31.49 | 14.85 |
| Procyanidin B-5 3,3'-di-O-gallate | [M+H]^+^ | 262915 | 180676 | 57631 | 0.246605 | 0.808 | 57.36 | 8.14 | -65.51 |
| Chrysin | [M+H]^+^ | 71225 | 2115911 | 63310 | 0.902033 | 0.288 | -90.51 | 182.07 | -91.56 |
| Quercetin 3-O-rutinoside-(1->2)-O-rhamnoside [Quercetin-3-O-(2G-alpha-L-rhamnosyl)-rutinoside] | [M+H]^+^ | 56928 | 6971890 | 365682 | 1.24287 | 0.871 | -97.69 | 182.85 | -85.16 |
| Caffeic acid | [M+H]^+^ | 166564 | 140657 | 319887 | 0.529849 | 0.408 | -20.32 | -32.71 | 53.03 |
| Resveratrol | [M+H]^+^ | 150390 | 702598 | 149991 | 0.352933 | 0.618 | -55.01 | 110.15 | -55.13 |
| 2,3,5,4'-Tetrahydroxyl diphenylethylene-2-O-glucoside | [M+H]^+^ | 35200 | 73242 | 752908 | 1.6807 | 0.393 | -87.74 | -74.49 | 162.23 |
| 5-Hydroxy-3',4',7-trimethoxyflavone | [M+H]^+^ | 2272898 | 718441 | 656172 | 0.939036 | 0.393 | 86.94 | -40.91 | -46.03 |
| Emodinanthrone | [M+H]^+^ | 273728 | 77938 | 1240076 | 0.471194 | 0.252 | -48.41 | -85.31 | 133.7205 |
| Keracyanin | [M+H]^+^ | 99073 | 1781486 | 152322 | 0.633983 | 0.639 | -85.38 | 162.91 | -77.52 |

Table 2 Comparison of peak areas and analysis of variable importance projection (VIP) values for BJP, DJP, and FJP compounds in negative ion mode

| Proposed phytochemicals | Adduct | Peak | | | VIP | P | Rate of change compared to the average (%) | | |
| --- | --- | --- | --- | --- | --- | --- | --- | --- | --- |
|  |  | BJP | DJP | FJP |  |  | BJP | DJP | FJP |
| Rosmarinic acid | [M+HCOO]^-^ | 50181 | 4588222 | 229480 | 0.554925 | 0.288 | -96.91 | 182.76 | -85.86 |
| Epicatechin | [M+HCOO]^-^ | 251041 | 203650 | 106831 | 1.00903 | 0.491 | 34.12 | 8.80 | -42.92 |
| Quercetin 3-O-neohesperidoside | [M-H]^-^ | 3369646 | 152211 | 63062 | 0.918562 | 0.097 | 181.99 | -87.26 | -94.72 |
| Rhein | [M-H]^-^ | 212215 | 127024 | 58233 | 0.313677 | 0.027 | 60.17 | -4.13 | -56.05 |
| Physcion | [M+HCOO]^-^ | 810226 | 1720585 | 96820 | 0.532535 | 0.576 | -7.49 | 96.44 | -88.95 |
| Quercetin 7-O-(6''-O-malonyl)-beta-D-glucoside | [M-H]^-^ | 29041 | 45421 | 849177 | 1.34157 | 0.142 | -90.56 | -85.24 | 175.81 |
| 7,3',4'-Trihydroxyflavone | [M+HCOO]^-^ | 2146937 | 268146 | 134109 | 0.508125 | 0.621 | 152.66 | -68.44 | -84.22 |
| Kaempferol | [M-H]^-^ | 269316 | 376170 | 39162 | 0.327728 | 0.361 | 18.01 | 64.83 | -82.84 |
| Galbinic Acid | [M+HCOO]^-^ | 2669498 | 2121109 | 1468618 | 0.554925 | 0.288 | 27.94 | 1.66 | -29.61 |
| Quercitrin | [M+HCOO]^-^ | 3629690 | 3059868 | 6510066 | 1.51242 | 0.067 | -17.50 | -30.46 | 47.96 |
| Syringic acid | [M-H]^-^ | 528776 | 5410178 | 6291091 | 1.96201 | 0.027 | -87.03 | 32.71 | 54.32 |
| trans- 4 - Coumaric acid | [M-H]^-^ | 55160 | 561528 | 532480 | 0.262771 | 0.409 | -85.6 | 46.59 | 39.01 |
| Procyanidin B3 | [M+HCOO]^-^ | 17852 | 30192 | 77338 | 1.06426 | 0.166 | -57.29 | -27.76 | 85.05 |
| Chrysin | [M+HCOO]^-^ | 573968 | 172520 | 63310 | 0.670869 | 0.046 | 112.63 | -36.09 | -76.54 |
| Caffeic acid | [M+HCOO]^-^ | 902016 | 1925555 | 2813635 | 1.0558 | 0.004 | -52.03 | 2.40 | 49.63 |
| Resveratrol | [M+HCOO]^-^ | 149528 | 58493 | 56829 | 0.40846 | 0.67 | 69.37 | -33.74 | -35.63 |
| 2,3,5,4'-Tetrahydroxyl diphenylethylene-2-O-glucoside | [M+H]^+^ | 3346545 | 7219302 | 383001 | 2.1779 | 0.066 | -8.30 | 97.81 | -89.51 |
| 5-Hydroxy-3',4',7-trimethoxyflavone | [M+HCOO]^-^ | 5506312 | 914485 | 10508448 | 1.85869 | 0.156 | -2.42367 | -83.7946 | 86.22 |
| Emodinanthrone | [M-H]^-^ | 38085 | 119056 | 31710 | 0.481872 | 0.091 | -39.4999 | 89.12688 | -49.63 |

1. ^*^Corresponding author: Junlan Li, E-mail address: qwzhaohui@126.com.
